# Augmenting Insufficiently Accruing Oncology Clinical Trials Using Generative Models: Validation Study

**DOI:** 10.2196/66821

**Published:** 2025-03-05

**Authors:** Samer El Kababji, Nicholas Mitsakakis, Elizabeth Jonker, Ana-Alicia Beltran-Bless, Gregory Pond, Lisa Vandermeer, Dhenuka Radhakrishnan, Lucy Mosquera, Alexander Paterson, Lois Shepherd, Bingshu Chen, William Barlow, Julie Gralow, Marie-France Savard, Christian Fesl, Dominik Hlauschek, Marija Balic, Gabriel Rinnerthaler, Richard Greil, Michael Gnant, Mark Clemons, Khaled El Emam

**Affiliations:** 1 School of Epidemiology and Public Health Faculty of Medicine University of Ottawa Ottawa, ON Canada; 2 CHEO Research Institute Ottawa, ON Canada; 3 Division of Medical Oncology University of Ottawa Ottawa, ON Canada; 4 Department of Oncology McMaster University Hamilton, ON Canada; 5 Generate Ops & Data Science Aetion Ottawa, ON Canada; 6 Oncology Alberta Health Services Edmonton, AB Canada; 7 Public Health Sciences Queens University Kingston, ON Canada; 8 Biostatistics University of Washington Seattle, WA United States; 9 Medical Oncology University of Washington Seattle, WA United States; 10 Clinical Statistics Austrian Breast & Colorectal Cancer Study Group (ABCSG) Vienna Austria; 11 Division of Clinical Oncology Medical University Graz Graz Austria; 12 Paracelsus Medical University Salzburg Salzburg Austria; 13 Comprehensive Cancer Center Medical University of Vienna Vienna Austria

**Keywords:** generative models, study accrual, recruitment, clinical trial replication, oncology, validation, simulated patient, simulation, retrospective, dataset, patient, artificial intelligence, machine learning

## Abstract

**Background:**

Insufficient patient accrual is a major challenge in clinical trials and can result in underpowered studies, as well as exposing study participants to toxicity and additional costs, with limited scientific benefit. Real-world data can provide external controls, but insufficient accrual affects all arms of a study, not just controls. Studies that used generative models to simulate more patients were limited in the accrual scenarios considered, replicability criteria, number of generative models, and number of clinical trials evaluated.

**Objective:**

This study aimed to perform a comprehensive evaluation on the extent generative models can be used to simulate additional patients to compensate for insufficient accrual in clinical trials.

**Methods:**

We performed a retrospective analysis using 10 datasets from 9 fully accrued, completed, and published cancer trials. For each trial, we removed the latest recruited patients (from 10% to 50%), trained a generative model on the remaining patients, and simulated additional patients to replace the removed ones using the generative model to augment the available data. We then replicated the published analysis on this augmented dataset to determine if the findings remained the same. Four different generative models were evaluated: sequential synthesis with decision trees, Bayesian network, generative adversarial network, and a variational autoencoder. These generative models were compared to sampling with replacement (ie, bootstrap) as a simple alternative. Replication of the published analyses used 4 metrics: decision agreement, estimate agreement, standardized difference, and CI overlap.

**Results:**

Sequential synthesis performed well on the 4 replication metrics for the removal of up to 40% of the last recruited patients (decision agreement: 88% to 100% across datasets, estimate agreement: 100%, cannot reject standardized difference null hypothesis: 100%, and CI overlap: 0.8-0.92). Sampling with replacement was the next most effective approach, with decision agreement varying from 78% to 89% across all datasets. There was no evidence of a monotonic relationship in the estimated effect size with recruitment order across these studies. This suggests that patients recruited earlier in a trial were not systematically different than those recruited later, at least partially explaining why generative models trained on early data can effectively simulate patients recruited later in a trial. The fidelity of the generated data relative to the training data on the Hellinger distance was high in all cases.

**Conclusions:**

For an oncology study with insufficient accrual with as few as 60% of target recruitment, sequential synthesis can enable the simulation of the full dataset had the study continued accruing patients and can be an alternative to drawing conclusions from an underpowered study. These results provide evidence demonstrating the potential for generative models to rescue poorly accruing clinical trials, but additional studies are needed to confirm these findings and to generalize them for other diseases.

## Introduction

### Background

Recruiting a sufficient number of patients for clinical trials is challenging [1], and the inability to recruit participants is the cause of failure for many clinical trials [2]. Approximately, 25% of clinical trials are discontinued before completion [3], with insufficient recruitment being the most frequent reason in 31% of the cases [4]. For adult cancer trials, between 20% and 50% fail to complete or were unable to reach recruitment goals [5-9]. This has been exacerbated by the recent pandemic where many trials experienced a considerable reduction in recruitment rates [10-13], which has continued after the pandemic [12]. While poor accrual is a problem in all trials, it is a greater problem in government (ie, academic) sponsored trials [14,15].

When a study is unable to recruit a sufficient number of patients, the study can be stopped, and the relevant analyses are performed on the available data. However, not reaching accrual targets results in underpowered analyses, and the smaller sample sizes increase the risk of unstable parameter estimates.

Patients have an expectation that their trial participation will lead to some advancement in knowledge that can be beneficial to the community [16], but many enroll in trials that do not answer the primary question adequately [15]. They are, therefore, enrolled in a study and exposed to toxicity and additional costs, with limited scientific benefit, which is considered unethical [17]. In addition to the wasted resources, it also means that those resources were not used for other studies that could have produced useful results.

Data augmentation is one approach to address insufficient accrual by either using real-world data (RWD) or by simulating additional observations.

RWD can be used for matched controls [18] where patient data from external sources are used instead of recruiting patients to the trial itself. In such a case, previous similar trials, registries, or eHealth record datasets on patients under the standard of care are matched to the treatment arm patients, and the matched patients’ data are used as the control arm [19,20]. Such an approach with external controls has been used for running single-arm oncology trials [21,22]. However, external controls are challenging for a number of reasons [18]. First, the patients from RWD may have different observed and unobserved characteristics than the treatment arm patients, despite the use of matching. Second, unaccounted for environmental factors, such as seasonal effects, may lead to outcome differences. Third, changes in medical practice may have occurred over time and since the external control data were collected. Fourth, there may be measurement differences between the treatment and external controls resulting in the pooling of incompatible datasets, even for objective metrics. Fifth, if there are no adequate matches in the external data for some of the treatment arm patients, then these treatment arm patients may need to be dropped resulting in loss of valuable data. Sixth, the outcome variables need to be available in the external control dataset to allow a comparison, which can be challenging for surrogate end points or patient-reported outcomes. Finally, and specific to our context, insufficient accrual would occur in all arms in a study and not only in the controls; therefore, external controls would not address our problem.

Augmentation through simulation can be a potential solution when there are insufficient data and is a common practice for imaging data [23-25] and time series data [26,27]. In the case of cross-sectional RWD, augmentation methods, such as sampling with replacement (henceforth referred to as *bootstrap*), and generative models, such as sequential synthesis using decision trees, generative adversarial networks (GANs), and variational autoencoders (VAEs), have been evaluated with encouraging results [28-32]. Augmentation methods have also been applied to small clinical trial datasets as a first step in synthetic data generation [33,34]. One study used a VAE generative model to simulate additional patients as a mechanism to design smaller clinical trials [35].

### Objective

In this study, we therefore adopt augmentation through simulation and expand on this body of work by more comprehensively evaluating multiple types of generative models and on a larger number of clinical trials. We make 2 hypotheses that we evaluate in this work as follows: (1) patients recruited early in a clinical trial are similar on the treatment effect to patients recruited later in a clinical trial and (2) because of hypothesis 1, we can train a generative model on early patients to simulate the remaining patients in insufficiently accruing trials to reach target recruitment and replicate the results of the original study that reached target recruitment.

Specifically, generative models are used to augment breast cancer clinical trials that do not reach target recruitment. We start with datasets from 9 completed breast cancer clinical trials and simulate different levels of insufficient accrual, and in each case, use generative machine learning models to simulate patients to compensate for the insufficient accrual. We then replicate the analyses of the published studies using the augmented datasets to determine if they produce similar findings as if the target number of patients were actually recruited.

## Methods

### Overview

Data augmentation methods using generative models were applied on 9 breast cancer clinical trial datasets. Insufficient accrual was simulated and augmentation then applied to compensate for that. The question was whether this can produce similar findings to the published analyses with the full data.

### Datasets

The clinical trials that were included are summarized in Table 1, with further details described in Table S1 in Multimedia Appendix 1 [36-38]. The Rethinking Clinical Trials (REaCT) were supported by the REaCT program at the Ottawa Hospital [39]. The remaining datasets included a larger number of patients and multiple sites.

Table 2 shows the countries that the patients were recruited from. The studies spanned multiple jurisdictions in North America, Europe, Australia, and South Africa.

**Table 1:** Key features of the clinical trial datasets used in this study.

Dataset	NCT identifier	Participants, N	Variables in dataset^a^, n	Strata info?	Control arm	Patients in the control arm, n (%)	Treatment arms considered	Patients in the treatment arm, n (%)	Enrollment period
REaCT^b^-ILIAD	NCT02861859	218	8	True	Placebo	105 (48)	Active olanzapine	113 (52)	December 2016 to June 2019
REaCT-BTA^c^	NCT02721433	230	8	True	4 weekly BTA	118 (51)	12 weekly BTA	112 (49)	August 3, 2016, to June 5, 2018
CCTG^d^ MA27	NCT00066573	7576	25	True	Anastrozole	3787 (50)	Exemestane	3789 (50)	June 2, 2003, to July 31, 2008
NSABP^e^ B34	NCT00009945	3310	49	True	Placebo	1656 (50)	Clodronate	1654 (50)	January 22, 2001, to March 31, 2004
REaCT-G/G2	NCT02428114 and NCT02816164	401	10	True	7 or 10 d of granulocyte colony stimulating factor	248 (62)	5 d of granulocyte colony stimulating factor	153 (38)	May 2015 to September 2018
REaCT-HER2+^f^	NCT02632435	50	47	True	Peripherally inserted central catheter	26 (52)	PORT; totally implanted vascular access device	24 (48)	March 2016 to March 2018
ABCSG^g^-12	NCT00295646	1803	35	False	Tamoxifen^h^	900 (50)	Anastrozole^h^	903 (50)	1999 to 2006
REaCT-ZOL^i^	NCT03664687	211	11	False	—^j^	—	—	—	November 1, 2018, to April 2, 2020
SWOG^k^ 0307^l^	NCT00127205	6018	23	False	Clodronate	2268 (38)	Zoledronic acid	2262 (38)	January 2006 to February 2010

^a^This is the total number of variables that were included in the generative models or bootstrap.

^b^REaCT: Rethinking Clinical Trials.

^c^BTA: bone-targeted agents.

^d^CCTG: Canadian Cancer Trials Group.

^e^NSABP: National Surgical Adjuvant Breast and Bowel Project.

^f^HER2+: human epidermal growth factor receptor-2 positive.

^g^ABCSG: Austrian Breast and Colorectal Cancer Study Group.

^h^Tamoxifen represents the arms of Nolvadex/control and Nolvadex/zoledronate in the clinical trial while anastrozole represents the arms of Arimidex/control and Arimidex/zoledronate.

^i^ZOL: zoledronate.

^j^Not applicable.

^k^SWOG: Southwest Oncology Group.

^l^Initially, the trial included 3 arms; however, only the indicated 2 arms had patients assigned to them throughout the duration of the study. Moreover, the available data for our study did not include any randomization codes, and as such, the original primary analysis comparing the outcomes between the 2 arms could not be replicated. Instead, we compared the 5-year survival probabilities between those with negative and positive or equivocal HER2 status. The estimate of the difference of survival probabilities and SE were produced.

**Table 2:** Countries of recruitment for the clinical trials used in this study.

Dataset	Countries of recruitment
REaCT^a^-ILIAD	Canada
REaCT-BTA^b^	Canada
CCTG^c^ MA27	Australia, Canada, Hungary, Italy, Puerto Rico, South Africa, Switzerland, and the United States
NSABP^d^ B34	United States
REaCT-G/G2	Canada
REaCT-HER2+^e^	Canada
ABCSG^f^-12	Austria and Germany
REaCT-ZOL^g^	Canada
SWOG^h^0307	Canada and the United States

^a^REaCT: Rethinking Clinical Trials.

^b^BTA: bone-targeted agents.

^c^CCTG: Canadian Cancer Trials Group.

^d^NSABP: National Surgical Adjuvant Breast and Bowel Project.

^e^HER2+: human epidermal growth factor receptor-2 positive.

^f^ABCSG: Austrian Breast and Colorectal Cancer Study Group.

^g^ZOL: zoledronate.

^h^SWOG: Southwest Oncology Group.

### Ethical Considerations

This study was a secondary analysis of datasets from already completed clinical trials. The secondary analysis was approved by the Children’s Hospital of Eastern Ontario Research Ethics Board (protocol: 23/47X) and the Ontario Cancer Research Ethics Board (project ID: 3749).

### Nonmonotonic Treatment Effect Size Hypothesis

We will use the terms “early participants” to indicate participants who were recruited in the earlier stages of a study and “late participants” to indicate those who were recruited in later stages of a study. For early participants to be good candidates for training a generative model that can be used to simulate late participants, there should not be a systematic difference in the estimated effect size between these 2 groups.

Estimated effect sizes tend to vary as patients are recruited and converge to the true value as more information is collected [40]. Instability of estimates at small sample sizes is a contributing factor. This means that training a generative model on earlier patients may enable the simulation of realistic patients that are representative of those that would be recruited later in the trial if the effect over time is not systematic.

However, existing sites gain experience with conducting a trial and this may result in process adjustments along that learning curve that may have an impact on the outcome. This can also happen, for example, when there is treatment effect heterogeneity whereby some patient characteristic (eg, disease severity or age) interacts with the intervention and more patients at one end of the severity or age scale are recruited earlier in the study [41]. One simulation demonstrated a monotonic change in effect size as more patients are recruited [42]. In that example, earlier patients had a meaningfully different estimated effect size compared to later patients with a trend over time. In such a case, training a generative model on earlier patients may not produce simulated patients that are representative of the later patients.

Therefore, it is an empirical question whether such a monotonic effect can be observed in practice.

As can be seen in Table 1, our 9 studies were conducted over an extended period. If the studies were very short, then there is a higher likelihood that early and late participants would be similar. However, the extended enrollment periods suggest that there was ample opportunity for the characteristics of the participants to change over time, as well as adjustments to the trial processes to occur as study staff gain more experience with time.

To test the hypothesis that early and late participants are similar on the estimated effect size, the monotonic relationship of the effect size and order of participant recruitment was examined with a regression model of treatment and recruitment order main effects and their interaction. Point estimates and 95% CIs of the interaction term were obtained, indicating statistical significance at *P*<.05 if they do not include 0. This investigation was not conducted for the trials where the main analysis did not use a statistical model with treatment as predictor (ie, REaCT-zoledronate [ZOL], Southwest Oncology Group 0307, and REaCT–human epidermal growth factor receptor-2 positive [HER2+]). If the effect size was not monotonic, then the interaction term would not be statistically significant.

### Generative Modeling Methods

#### Overview

We used 4 common machine learning–based generative modeling methods for structured tabular data to synthesize the analysis datasets from the clinical trials under investigation. These methods include sequential synthesis using decision trees, Bayesian networks, GAN, and VAE. The last 3 methods were implemented as an adaptation of an open-sourced Python package *Synthcity* [43]. Our implementation, which is publicly available (*Data Availability* section), provides further preprocessing and postprocessing on top of *Synthcity*. No further hyperparameter tuning was performed beyond what was available in the *Synthcity* implementation. For each generative model, the number of variables indicated in Table 1 were synthesized.

In addition, we used the “bootstrap” technique for comparison as a baseline. Bootstrap is simply sampling with replacement from the training data to add the missing patients.

#### Sequential Decision Trees

Similar to using a chaining method for multilabel classification problems, sequential decision trees generate synthetic data using conditional trees in a sequential fashion [44-46]. It has been commonly used in the health care and social-science domains for data synthesis [28,47-54]. The details of the implementation procedures are described elsewhere [44].

#### Bayesian Networks

Bayesian networks are models based on directed acyclic graphs that consist of nodes representing the random variables and arcs representing the dependencies among these variables. To construct the Bayesian networks model, the first step is to find the optimal network topology and then to estimate the optimal parameters [55]. Starting with a random initial network structure, the Hill Climb heuristic search is used to find the optimal structure. Then, the conditional probability distributions are estimated using the maximum a posteriori estimator [56]. Once the network structure and the parameters are estimated, we can initialize the nodes with no incoming arcs by sampling from their marginal distributions and predict the rest of the connected variables using the estimated parameters.

#### Conditional GAN

A basic GAN consists of 2 artificial neural networks, a generator and a discriminator [57]. The generator and the discriminator play a min-max game. The input to the generator is noise, while its output is synthetic data. The discriminator has 2 inputs: the real training data and the synthetic data generated by the generator. The output of the discriminator indicates whether its input is real or synthetic. The generator is trained to “trick” the discriminator by generating samples that look real. In contrast, the discriminator is trained to maximize its discriminatory capability.

Among all the variations of GAN architectures, the conditional tabular GAN (CTGAN) is often used in tabular data synthesis [58]. CTGAN builds on conditional GANs by addressing the multimodal distributions of continuous variables and the highly imbalanced categorical variables [59]. CTGAN solves the first problem by proposing a per-mode normalization technique. For the second problem, each category of a categorical variable serves as the condition passed to the GAN.

#### Variational Autoencoder

VAEs use artificial neural networks and involve 2 steps (ie, encoding and decoding) to generate new samples [60]. First, an encoder is generated to compress input data into a lower-dimensional latent space, in which the data points are represented by distributions. The second step is a decoding process, in which new data samples are reconstructed as output from the latent space. The neural network is optimized by minimizing the reconstruction loss between the output and the input. VAEs are known to generate complex data of various types due to their ability to learn more complex distributions and relationships [61]. Many variants have been proposed as an extension of VAE, such as triplet-based VAE [62], conditional VAE [63], and Gaussian VAE [64]. In particular, the tabular VAE was proposed as an adaptation of standard VAE to model and generate mixed-type tabular data with a modified loss function [59].

### Augmentation

The augmentation procedure ensured that the augmented data had the same number of patients as the original clinical trial. Therefore, there is no difference in size between the published study and the augmented datasets used in our analyses.

**Figure 1:**
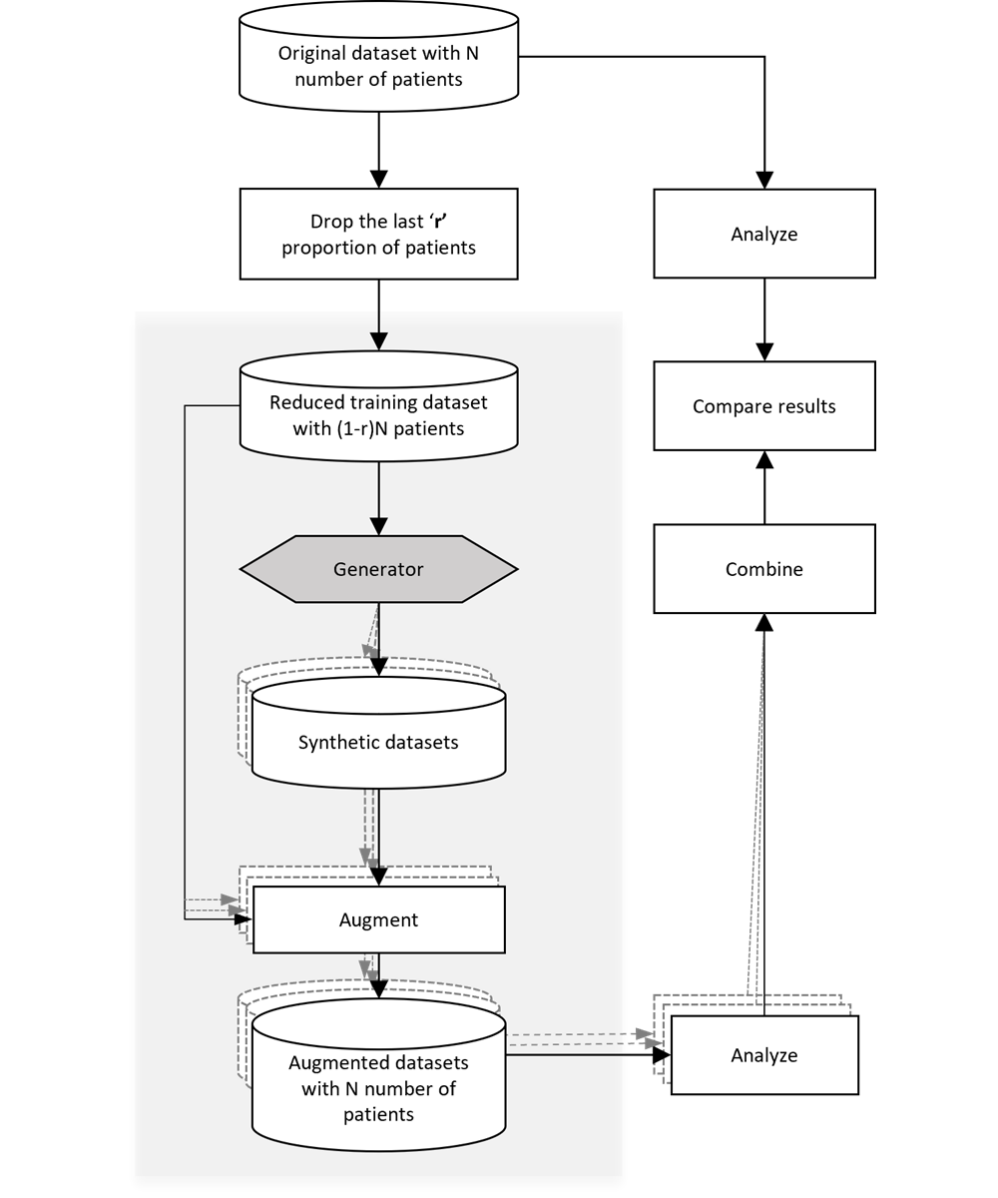
Augmentation of clinical trial datasets using the generative model.

Figure 1 illustrates the main steps of generating the augmented datasets for the trials given in Table 1. A trial’s *original* dataset, with N number of patients, was first reduced by *r*. The variable *r* signifies the fraction of the *last* patients who were deliberately removed from the input dataset. This results in a *reduced* dataset with “(1-r) N” patients. In practice, the reduced dataset represents a poorly accruing clinical trial that needs to be rescued. The shaded area outlines the typical steps taken by a practitioner during the implementation process.

In our study, the *r* value was varied incrementally from 0.1 to 0.5 in steps of 0.1. For instance, a 0.2 *r* value indicates that the last 20% of patients were deliberately removed from the original trial dataset. The reduced dataset was then used to train a generative model. In case of bootstrapping, the reduced dataset was used for sampling with replacement. After training, the necessary samples for augmentation were generated from the trained model and concatenated onto the reduced dataset. To account for stochasticity, and as depicted by the dotted lines in the Figure 1, ten versions of the synthetic data were generated leading to multiple versions of the augmented datasets. Subsequently, each of the augmented datasets were analyzed in the same way as the published analysis. The 10 analyses results were then combined to obtain a single augmented dataset result, and that was compared with the results obtained from the original dataset (as described in the subsequent section).

Some of the trials had implemented stratified randomization, and in those cases, we modified the basic process to accommodate stratification, for example, consider that as per the predefined protocol, the original dataset is stratified by 2 variables, the “Cancer Type” and the “Site Number.” Rejection sampling was used to draw from the generated datasets to achieve the desired strata proportions along these 2 dimensions, in addition to the appropriate numbers of patients in each arm of the study.

### Combining Rules

The original proposal for synthetic data generation treated it as a form of multiple imputation [65]. Under the multiple imputation model, multiple datasets, say *m*, are synthesized and analyzed. The combining rules are used to compute the parameter estimates and variances across the analysis results from the *m* synthetic datasets [66-68]. Such corrections for the parameter estimates and variances ensured that variability introduced by the generative process are accounted for when estimating parameters and making population inferences from synthetic datasets.

Once the generative model was trained on the underlying distribution of the input data, it was used to create *m* synthetic datasets of size *r* × N, and then each was added to that training dataset to augment it. This resulted in *m* versions of augmented datasets, each containing N records.

The *m* augmented datasets were analyzed using the same methodology applied to the original data in the relevant publications. This analysis yielded estimated parameters for each of the augmented versions. Subsequently, these estimated parameters were combined in accordance with the following partial synthesis rules.

For a particular model parameter *q_i_* with variance *v_i_* using synthetic dataset *i* where *i=1...m*. The adjustment for the model parameters and variances is as follows [52,68,69]. The combined model parameter 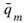. is the mean across the m model parameters from the synthetic datasets 
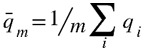
, and 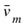 is the mean variance across the *m* model parameters from the synthetic datasets where 
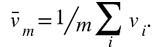
. The between imputation variance is given by 
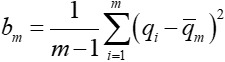
, the adjusted variance is computed as 
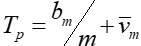
, and the adjusted large sample 95% CI of the model parameter is computed as 
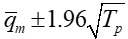
. For this study, we set *m*=10, which is consistent with current practice for the analysis of synthetic data [52,68-70] and has been recommended based on a recent simulation [71].

### Augmentation Fidelity

For the generated datasets, we evaluated the fidelity relative to the training dataset. Fidelity indicates the extent to which the distributions of generated data deviate from the training data. For example, if *r*=0.1, then 90% of the dataset is used for training the generative model, and 10% is generated. We assessed the fidelity using the Hellinger distance [72], which has the advantage of being interpretable as it varies from 0 to 1. The Hellinger distance is averaged across the 10 generated datasets.

### Evaluation of Study Replicability

We evaluated the replicability of the analysis results using the augmented datasets. Replicability is the reliability of findings when an existing study is repeated using the same analytical methods but different data [73]. We assessed it by comparing the published analysis results using the real datasets for these clinical trials with the results of the same analysis performed on the partially synthetic (ie, augmented) data. The details of the published analyses that were replicated are summarized in Multimedia Appendix 1.

For each clinical trial, a model was fitted to obtain a parameter estimate and its SE. For instance, if generalized estimating equations [36] were used for modeling the real data, an estimate would be the coefficient associated with the selected predictor as per the published study. We obtained a value for the estimate and its 95% CI. The same was applied to each of the *m* versions of the augmented data. We combined the results from all the augmented versions using the combining rules discussed above. Subsequently, the estimates and CIs of the original and augmented data were compared in terms of the *estimate agreement*, the *decision agreement*, *standardized difference*, and the *CI overlap*. These criteria have been used in the literature to assess the replicability of analyses using synthetic data [71,74]. The criteria are defined in Textbox 1.

**Criteria to assess the replicability of analyses using synthetic data.**

- *Estimate agreement*: It is a Boolean indicator of whether the estimate produced by the augmented data is within the 95% CI produced by the real data. This requires that an augmented data effect estimate be within the range of plausible values for the true effect based on evidence from the real data. Under the assumption that the parameter variances are equal between the real and augmented datasets, estimate agreement is expected 83% of the time under no bias [75].
- *Decision agreement*: It is a Boolean indicator of whether the same conclusion is drawn from the real and augmented data estimates. This means that the augmented data estimates have the same direction and statistical significance as the real data. The decision agreement does not apply if the analysis is descriptive. We would expect decision agreement to occur at a rate equal to power, which would be at least 80% of the time (ie, assuming the 9 trials are powered by design for at least 80%) [75].
- *Standardized difference*: It is a Boolean indicator of whether the difference in the parameter estimate between real and augmented data is consistent with the null hypothesis of no difference [75]. The Z value is computed and compared with the standard normal (|Z|≤1.96).
- *CI overlap*: It is a proportion of the real and augmented data parameter CIs overlap [76], which is a commonly used synthetic data utility metric. We would want this to be as close to 100% as possible but set 80% as a minimal value.

The 2 agreement metrics are consistent with previous measures of replicability [77-79], have been used to compare RWD analysis results against a clinical trial reference [75,80-83], and have been used to assess the replicability of psychological studies [77].

## Results

The first set of results are shown in Table 3 where the monotonic relationship of the effect size over time is investigated. In none of the trials used for the study was the interaction term (ie, recruitment order by treatment) found to be statistically significant, indicating lack of evidence of monotonically varying treatment effect with respect to the order of participant recruitment.

**Table 3:** Evaluation of monotonic relationships over recruitment order in the estimated effect size.

Trial	Main effect (95% CI)	Interaction effect (95% CI)
REaCT^a^-ILIAD	0.52 (0.023 to 1.01)	−0.00000371 (−0.0000116 to 4.16×10^–6^)
REaCT-BTA^b^	−1.85 (−4.75 to 1.04)	0.011 (−0.032 to 0.0542)
CCTG^c^ MA27	0.033 (−0.117 to 0.18)	−0.0000135 (−0.0000854 to 5.84×10^–5^)
NSABP^d^ B34	−0.036 (−0.18 to 0.11)	8.28×10^–5^ (−0.000072 to 0.00024)
REaCT-G/G2	0.015 (−0.0019 to 0.032)	6.6×10^–6^ (−0.000005 to 1.85×10^–5^)
ABCSG^e^-12 (tamoxifen vs anastrozole)	0.11 (−0.13 to 0.35)	7.36×10^–5^ (−0.0004 to 0.00054)
ABCSG-12 (zoledronic acid vs no zoledronic acid)	−0.24 (0.48 to 0.002)	6.69×10^–5^ (−0.00041 to 0.00054)

^a^REaCT: Rethinking Clinical Trials.

^b^BTA: bone-targeted agents.

^c^CCTG: Canadian Cancer Trials Group.

^d^NSABP: National Surgical Adjuvant Breast and Bowel Project.

^e^ABCSG: Austrian Breast and Colorectal Cancer Study Group.

The results on the data with insufficient accrual (ie, the “reduced” datasets with no augmentation) for 2 values of *r* at 0.2 and 0.5 are shown in Table 4.

We can make 4 observations from Table 4:

1. For the REaCT-HER2+ study, which had the smallest sample size, there is no decision agreement as *r* increases. This is not surprising as the low sample sizes would mean unstable parameter estimates and larger CIs. The small sample size also explains the lack of difference in the standardized difference comparison.
2. The REaCT-ILIAD study had a statistically significant result with the full data. Decision agreement is no longer attained as *r* increases due to the smaller sample sizes, lower power, and hence, wider CIs.
3. The Austrian Breast and Colorectal Cancer Study Group (ABCSG)-ZOL analysis had a marginal nonsignificant outcome in the full trial. At small *r* values, small changes can have an impact on the statistical significance of the results, but that becomes less of an issue for large *r* values as the results will no longer be marginal with wider CIs. Although, one would expect that if the original results were marginally statistically significant, there would not be decision agreement even for larger values of *r*.
4. For the remaining trial analyses, the results were not significant in the original data. Therefore, the decision agreement would not be affected with the lower power as *r* increases, and the parameter estimates retained the same direction as the full trial. The standardized difference comparison indicates that the parameters were not different even as *r* increases, which is consistent with the inability to detect a monotonic effect with recruitment order presented above, and detecting a difference becomes more difficult as the sample size decreases.

These results indicate that drawing conclusions from reduced datasets can be misleading and can produce incorrect findings relative to those that would be obtained if target recruitment was achieved. In addition, the nature of any error would not be known a priori.

**Figure 2:**
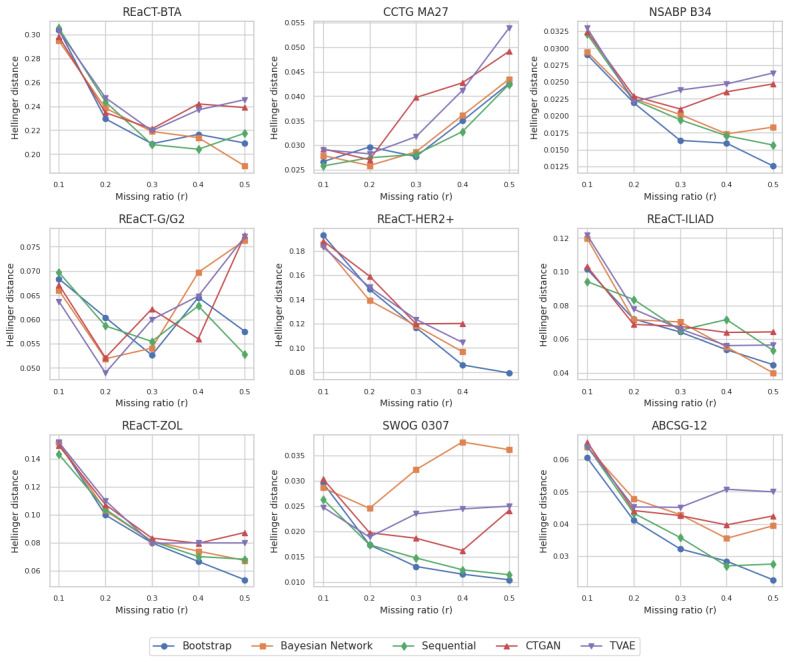
Hellinger distance results by comparing the training dataset with the generated dataset. The values were averaged across all generated 10 datasets. A value of 0 means that the 2 datasets are the same and of 1 indicates maximum difference. Note that the y-axis scales are not the same across all plots to provide better readability. ABCSG: Austrian Breast and Colorectal Cancer Study Group; BTA: bone-targeted agents; CCTG: Canadian Cancer Trials Group; CTGAN: conditional tabular generative adversarial network; HER2+: human epidermal growth factor receptor-2 positive; NSABP: National Surgical Adjuvant Breast and Bowel Project; REaCT: Rethinking Clinical Trials; SWOG: Southwest Oncology Group; TVAE: tabular variational auto encoder; ZOL: zoledronate.

The fidelity results for the augmented datasets are shown in Figure 2. At low values of *r*, the generated datasets were small, making fidelity comparisons unstable. For the smaller datasets, such as REaCT-HER2+, REaCT–bone-targeted agents (BTA), REaCT-ZOL, and REaCT-ILIAD, the Hellinger distance values were highest but still relatively low on an absolute scale. For the other datasets, the Hellinger values were quite small with variation in a very narrow range, demonstrating high fidelity.

**Table 4:** The baseline primary results for the original datasets, and for the reduced datasets at 2 different values of *r* (ie, 0.2 and 0.5)

Trial name	Trial short name	Full dataset				*r*=0.2							*r*=0.5					
		Effect size (SE)	Variables used in the analysis	Analysis method	Sample size, N		Effect size (SE)	Estimate agreement	Decision agreement	Standardized difference	CI overlap	Sample size, N		Effect size (SE)	Estimate agreement	Decision agreement	Standardized difference	CI overlap
REaCT^a^-ILIAD	ILIAD [84]	0.52 (0.25)	8	GEE^b^	174		0.64 (0.28)	1	1	1	0.88	109		0.39 (0.36)	1	0	1	0.85
REaCT-BTA^c^	BTA [85]	−1.85 (1.48)	8	LM^d^	184		−2.54 (1.63)	1	1	1	0.89	115		−3.46 (2.03)	1	1	1	0.79
CCTG^e^ MA27	CCTG [86]	0.033 (0.076)	6	Cox^f^	6060		0.045 (0.084)	1	1	1	0.96	3788		−0.006 (0.102)	1	1	1	0.87
NSABP^g^ B34	NSABP [87]	−0.036 (0.076)	6	Cox	2648		−0.034 (0.085)	1	1	1	0.95	1655		−0.124 (0.108)	1	1	1	0.78
REaCT-G/G2	G/G2 [88]	0.015 (0.009)	10	GEE	320		0.012 (0.010)	1	1	1	0.91	200		0.0005 (0.016)	1	1	1	0.76
REaCT-HER2+^h^	HER2+ [89]	0.0058 (0.0062)	3	GLM^i^	40		0.0075 (0.006)	1	1	1	0.93	25		0.017 (0.009)	1	0	1	0.62
ABCSG^j^-12 (tamoxifen vs anastrozole)	ABCSG [90]	0.11 (0.12)	3	Cox	1442		0.13 (0.14)	1	1	1	0.96	901		0.07 (0.17)	1	1	1	0.87
ABCSG-12 (ZOL^k^ vs no ZOL)	ABCSG-ZOL [90]	−0.24 (0.1237)	3	Cox	1442		−0.27 (0.14)	1	0	1	0.95	901		−0.26 (0.17)	1	1	1	0.87
REaCT-ZOL	ZOL [91]	58.98^l^ (0.76)	2	*t* test	168		58.07 (0.89)	1	—	1	0.72	105		58.20 (1.10)	1	—	1	0.81
SWOG^m^ 0307	SWOG [92]	−0.0087 (0.0145)	3	Surv^n^	4814		4.03×10^–5^ (0.016)	1	1	1	0.86	3009		−0.0096 (0.02)	1	1	1	0.86

^a^REaCT: Rethinking Clinical Trials.

^b^GEE: general estimating equations.

^c^BTA: bone-targeted agents.

^d^LM: linear model.

^e^CCTG: Canadian Cancer Trials Group.

^f^Cox: Cox regression

^g^NSABP: National Surgical Adjuvant Breast and Bowel Project.

^h^HER2+: human epidermal growth factor receptor-2 positive

^i^GLM; general linear model.

^j^ABCSG: Austrian Breast and Colorectal Cancer Study Group.

^k^ZOL: zoledronate.

**^l^**No effect was estimated for REaCT-ZOL—the figures shown correspond to descriptive analysis.

^m^SWOG: Southwest Oncology Group.

^n^Surv: difference in survival probabilities.

**Figure 3:**
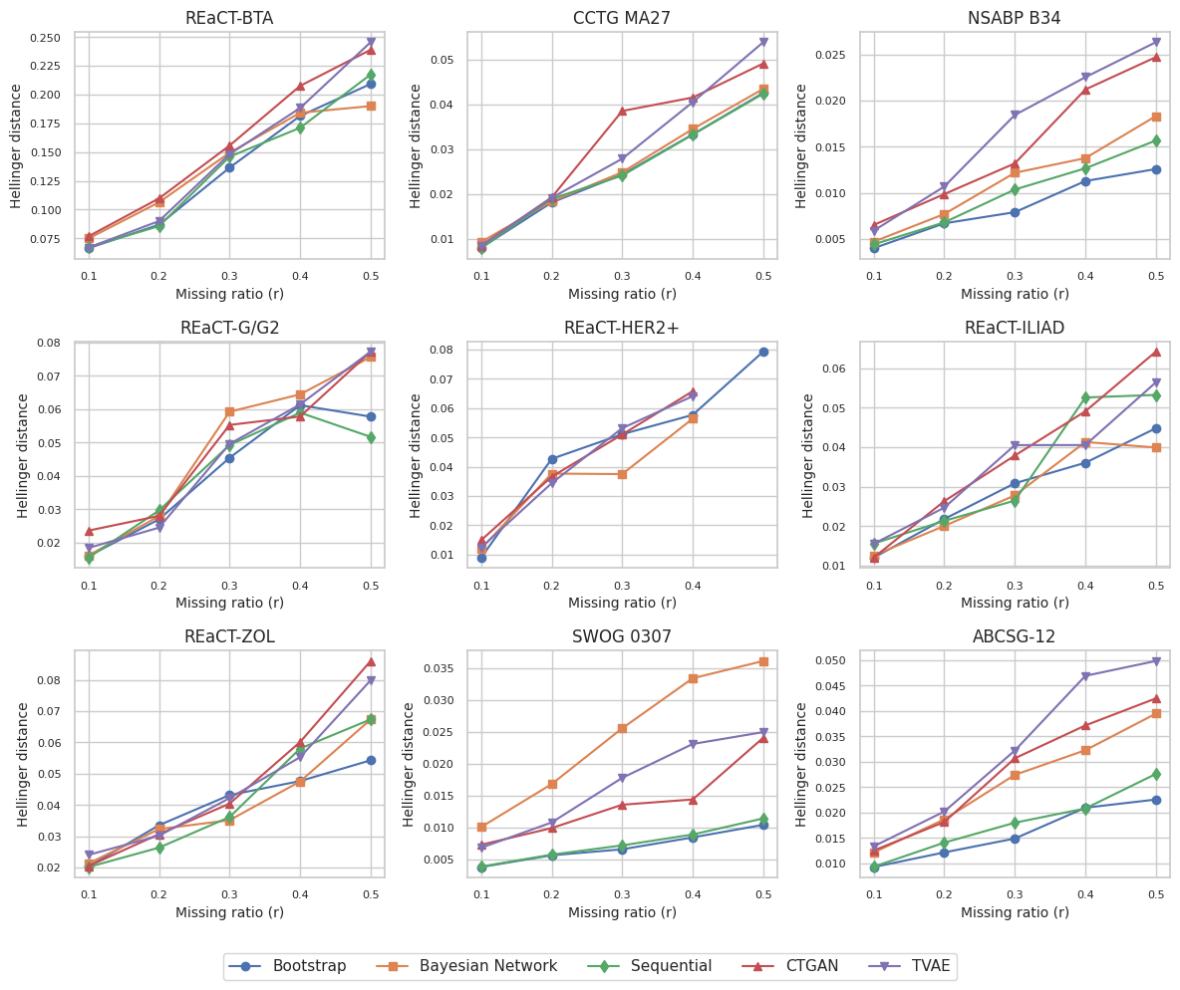
Hellinger distance results by comparing the training dataset with a generated dataset of the same size. The values were averaged across all generated 10 datasets. A value of 0 means that the 2 datasets are the same and of 1 indicates maximum difference. Note that the y-axis scales are not the same across all plots to provide better readability. ABCSG: Austrian Breast and Colorectal Cancer Study Group; BTA: bone-targeted agents; CCTG: Canadian Cancer Trials Group; CTGAN: conditional tabular generative adversarial network; HER2+: human epidermal growth factor receptor-2 positive; NSABP: National Surgical Adjuvant Breast and Bowel Project; REaCT: Rethinking Clinical Trials; SWOG: Southwest Oncology Group; TVAE: tabular variational auto encoder; ZOL: zoledronate.

Another representation of fidelity is shown in Figure 3, where we compared the training dataset with a generated dataset of the same size. Here, we can see high fidelity values for all datasets as these results were less affected by small dataset sizes. As the training dataset size decreased with higher *r*, the fidelity decreased, but the changes in fidelity were modest.

All the detailed replicability results for all values of *r* are provided in Table S2 in Multimedia Appendix 1.

**Figure 4:**
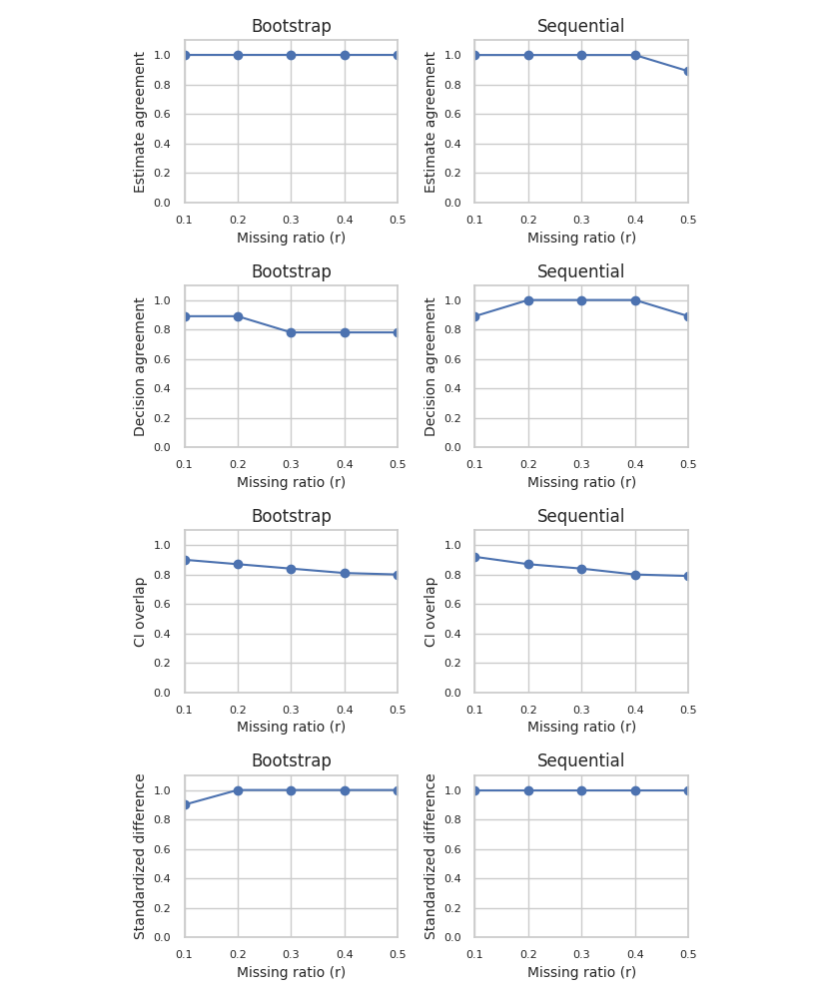
All 4 metrics calculated across all the datasets for the bootstrap and sequential generators. For estimate agreement, decision agreement, and standardized difference, the y-axis is the proportion across all datasets. For CI overlap, the y-axis is the average across all data sets. Modeling failures are not considered.

The first general observation is that the bootstrap and sequential synthesis tend to perform best among all the generative models. Therefore, in Figure 4 we only show their results across all datasets that were examined for the 4 measures of replicability. Both types of generative models achieved high estimate and decision agreement and maintained a CI overlap above 80% even as *r* values approached 0.5.

It should be noted that sequential synthesis fails for the smallest clinical trial and that was not included in the denominator for Figure 4. This failure was a design decision in the implementation that we used to not train a model with less than 50 observations. Also note that in the plots model failures are not counted in the denominator.

**Figure 5:**
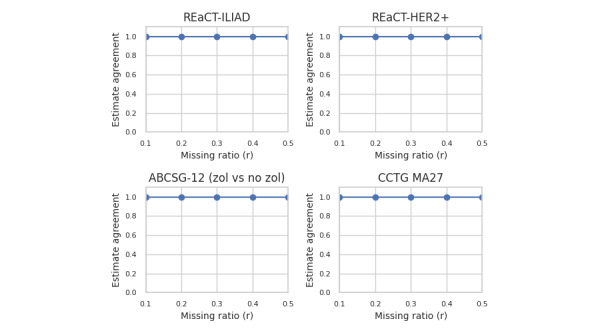
Estimate agreement for selected datasets—proportion across all generators. ABCSG: Austrian Breast and Colorectal Cancer Study Group; CCTG: Canadian Cancer Trials Group; HER2+: human epidermal growth factor receptor-2 positive; REaCT: Rethinking Clinical Trials; ZOL: zoledronate.

**Figure 6:**
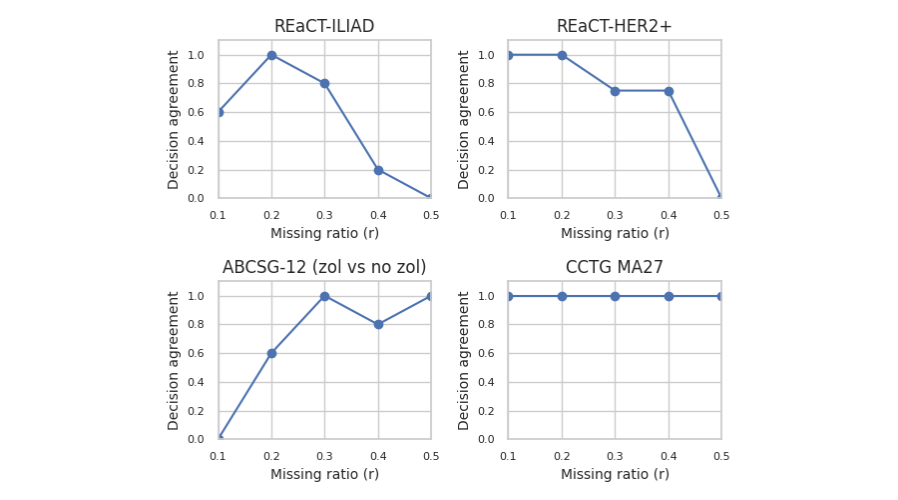
Decision agreement for selected datasets—proportion across all generators. ABCSG: Austrian Breast and Colorectal Cancer Study Group; CCTG: Canadian Cancer Trials Group; HER2+: human epidermal growth factor receptor-2 positive; REaCT: Rethinking Clinical Trials; ZOL: zoledronate.

**Figure 7:**
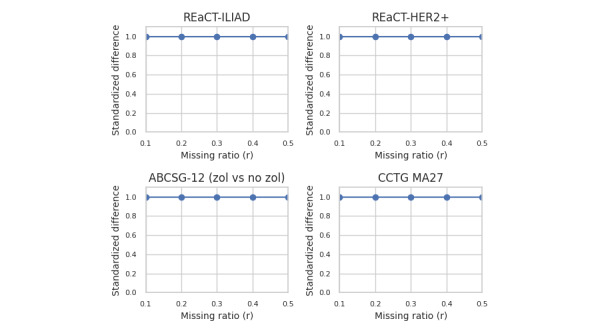
Standardized difference indicator for selected datasets—proportion across all generators. ABCSG: Austrian Breast and Colorectal Cancer Study Group; CCTG: Canadian Cancer Trials Group; HER2+: human epidermal growth factor receptor-2 positive; REaCT: Rethinking Clinical Trials; ZOL: zoledronate.

**Figure 8:**
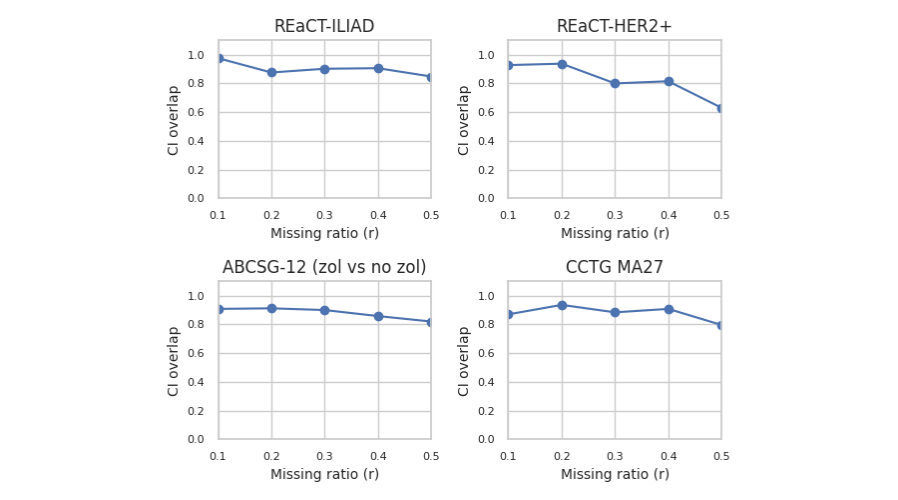
CI overlap for selected datasets—average across all generators. ABCSG: Austrian Breast and Colorectal Cancer Study Group; CCTG: Canadian Cancer Trials Group; HER2+: human epidermal growth factor receptor-2 positive; REaCT: Rethinking Clinical Trials; ZOL: zoledronate.

For the results by dataset, we focus on 4 trials that exemplify all the scenarios in our dataset across all 4 generative models, and are shown in Figure 5 for estimate agreement, Figure 6 for decision agreement, Figure 7 for the standardized difference, and Figure 8 for the CI overlap. The values were consistently high for estimate agreement, standardized difference, and CI overlap across all the scenarios, even at values of *r* approaching 0.5. The results for decision agreement in Figure 6 were more varied and can be characterized as follows:

1. For studies where there was a statistically significant result, for example, REaCT-ILIAD, the ability to maintain decision agreement deteriorates with higher values of *r*. The best performing generative model was sequential synthesis in that decision agreement was maintained for *r* as high as 0.4. Next was the bootstrap, which had a decision agreement for *r* up to 0.3.
2. For studies where the results were not statistically significant (eg, Canadian Cancer Trials Group MA27) or a marginal nonsignificant result (eg, ABCSG–ZOL), the value of *r* had no impact on the replicability of the results in that all the results were successfully replicated.
3. For smaller studies (eg, REaCT-ZOL), some generative models were able to maintain decision agreement for *r* as high as 0.3, although most models failed for *r* greater than that.

## Discussion

### Summary

Many clinical trials face accrual problems, resulting in an inability to reach target recruitment and resulting in analysts drawing conclusions from potentially underpowered studies. This exposes patients to toxicity and additional costs, with potentially no scientific benefit. Accrual problems may be due to genuine difficulty with recruitment of patients or with execution quality challenges during the trial itself.

When a study is unable to recruit more patients, the study can be stopped, and the relevant analyses is performed on the available data. For small trials, analyzing the data with insufficient accrual results in even smaller sample sizes, which can produce unstable parameter estimates and direction. For larger trials, when the complete trial results are statistically significant, an analysis with insufficient accrual can be underpowered and result in nonsignificant findings. For marginal results with the full data, insufficient accrual can reverse their statistical significance. When the full study results are not significant, then insufficient accrual would have less of an impact. A priori, it would not be known which one of these situations pertains to a particular study, making it difficult to interpret the results when analysis is performed with unplanned accrual deficiencies.

The objective of this study was to determine whether generative models can be a useful tool to rescue clinical studies that have insufficient accrual, through augmentation. Generative models have been applied to simulate participants [93-96] and counterfactuals [95,96] in the context of in silico clinical trials. While there have been concerns about generative models overfitting for the small datasets typically encountered in clinical trials [96,97], recent studies have been able to generate synthetic variants of full clinical trial datasets with high utility [74,95,97-102].

To test the ability of generative models to augment clinical trial datasets, we evaluated 4 different types of commonly used generative models (ie, sequential synthesis using decision trees, Bayesian network, GAN, and VAE) on 9 different breast cancer clinical trials and 10 different analyses. The study simulated different degrees of insufficient accrual and the generative models simulated replacement patients to compensate. The last fraction of recruited patients were replaced with simulated ones ranging from *r*=0.1 to *r*=0.5. In addition to generative models, we evaluated a bootstrap approach. These augmented datasets were then used to replicate the published analyses (ie, using the complete datasets) on these 9 trials.

An important assumption for these augmentation methods to work was that participants recruited early were not systematically different than late participants in their estimated effect size. It has been argued that estimated effect sizes tend to vary as patients are recruited and converge to the true value with more information [40,42]. In contrast, as sites gain experience and adjust their processes, there could be a monotonic treatment effect over recruitment time. Also, consider, for example, if there was treatment effect heterogeneity on disease severity (ie, the impact of the treatment on the outcome depends on disease severity), with high severity patients having a bigger response to the intervention, and fewer high severity patients were recruited early in the study compared with late in the study. This would manifest itself as a monotonic relationship between the estimated effect size and patients recruited over time.

We tested that monotonic relationship hypothesis by examining the interaction effect between recruitment order and treatment on the outcome. We did not find evidence across the trials that this relationship was monotonic (ie, all interaction effects were small and not statistically significant), meaning that we could not detect an increasing or decreasing effect size as more patients were recruited. This is despite these trials having long enrollment periods, in some cases lasting years. Therefore, if the early and late participants were, on average, similar in the estimated effect size, then generative models and a simple bootstrap would also be expected to work well.

These results are supportive of hypothesis 1, which stated that patients recruited early in a trial are similar to those recruited later in the trial.

Furthermore, the fidelity of the generated datasets was quite high relative to the training datasets from the different generative models. In all cases, the patterns in datasets that were synthesized had a high similarity on the Hellinger distance to the datasets of the participants already recruited.

Several observations can be made from our results:

1. A bootstrap would be attractive due to its simplicity and low computational burden. However, this method tended to lack consistent decision agreement when a trial result was statistically significant and when the trial was small, while in other scenarios, sampling with replacement performed well. However, the former are nontrivial failure modes.
2. All approaches struggled with marginal results (eg, marginally nonsignificant results) when the *r* value was low. This indicates a general sensitivity to that particular scenario.
3. For *r* values as high as 0.4, sequential synthesis performed well across all datasets. This means that both decision agreement and estimate agreement were achieved, CI overlap was at or above 0.8, and it was either equivalent to or better than the other methods evaluated.
4. Bayesian networks and the GAN had the next best performance after sequential synthesis; however, the Bayesian network had slightly better CI overlap overall up to an *r*=0.4.

To ensure reasonable performance across multiple scenarios, the results suggest that sequential synthesis can be used to address insufficient accrual up to *r*=0.4 (ie, only 60% of the target is recruited).

These results are supportive of hypothesis 2, which stated that generative models can simulate the remaining patients in a clinical trial with insufficient recruitment, and the augmented dataset would replicate the results if the trial did reach target recruitment.

It should be noted that using generative models to simulate additional patients as described in this study would not be preplanned, as opposed to a planned interim analysis. Insufficient accrual becomes a problem when there are no budgeted resources available to continue recruiting patients, for example, by adding sites, extending recruitment time, or changing the inclusion and exclusion criteria. Although, if the results from the augmentation show positive findings, the case may be made to allocate more resources to continue recruitment. Additionally, if the results from augmentation show negative findings, then that would provide a stronger case for terminating the study.

The countries of recruitment for the clinical trials used in this study cover multiple regions around the world as shown in Table 2. Our results were consistent across the different jurisdictions. Therefore, it would be reasonable to have confidence that the findings are generalizable across multiple jurisdictions and not specific to a particular region.

### Comparison With Prior Work

Previous studies have demonstrated that sequential synthesis performs well (ie, in terms of replicability of published studies) on oncology clinical trial datasets [74,98], and therefore, our findings are consistent with that evidence. More generally, sequential synthesis has been found to have superior utility across different types of datasets relative to other types of generative models [103-105]. Also, it should be noted that most of the published analyses that were replicated across all clinical trials used datasets that were low dimensional, which imposes lower sample size requirements for the generative models.

An earlier study found that a VAE generative model trained on early patients could augment a clinical trial with simulated patients [35]. In that study, the authors argued that generative models can also enable the design of smaller studies to start off with. This means that studies would be designed to be smaller, with augmentation used to reach target recruitment for the final analysis. However, that analysis only considered a single simulated clinical trial (ie, not real data).

The argument for using augmentation to prospectively design smaller studies is appealing. The largest factor driving up the cost of trials is the number of participants required to achieve sufficient statistical power [106,107]. The median cost per participant in drug trials (ie, in general) was estimated to be US $41,413 [107], the median cost per participant specifically in oncology drug trials was US $100,271 [107], and an earlier study found the 1-year cost to be US $17,003 per patient in the treatment arm and US $15,516 per control participant [108]. Designing studies that require fewer patients to be recruited can improve the cost-effectiveness of studies.

Our current analysis generalizes this work to other types of generative models, including a VAE, but we did find that a VAE did not perform well on our criteria. Plus, we performed the evaluation on 9 real clinical trial datasets of different sizes and durations and compared the generative models to a simple bootstrap. Furthermore, an important difference is that rescuing a study due to insufficient accrual using augmentation is not planned, whereas designing a small study is planned.

Nevertheless, using augmentation to design smaller studies deserves further investigation, and it remains a necessity that researchers aim to recruit the target sample size wherever possible.

Clinical trials are known to underrepresent certain groups, and hence, there is the potential for introducing bias in the results. For instance, a recent study in Canada found that the underrepresentation of Black patients in cancer research remains a significant concern, with 15 out of the 20 most common types of cancer not being studied in Black communities [109,110]; studies on underrepresented populations in clinical trials show racial and ethnic disparities worldwide [111-118]; and there is a consistent underrepresentation of various other groups, such as older adults [111,112,119-122], women [111,113,119,120,123], and individuals of lower socioeconomic status and educational level [111,112,124]. Furthermore, synthetic data generation has been shown to introduce bias in the generated data relative to the training data [125], and these biases are propagated across multiple generations of generative models, where the output of one is used as training for the next one [126].

Our analysis did not explicitly evaluate representation bias as we were replicating the published analyses rather than identifying and correcting any weaknesses and did not explicitly attempt to mitigate such underrepresentation to the extent that it existed in the original datasets. Nevertheless, analysts can also apply augmentation methods to compensate for any biases that may exist in the training datasets [127-129].

Another risk of bias is if there is a relationship between a particular characteristic and the order of recruitment. For example, if the first 60% of patients recruited were aged mostly >70 years and the last 40% were aged mostly <70 years, then the trained generative model and their simulated patients would not include sufficient younger patients. However, such an age bias would only impact the randomized study outcomes if there was an interaction between recruitment order, or factors correlated to it, such as age in this example, and the treatment. We explicitly tested for such an interaction effect, and as seen in the results, there were none detected.

Generative models can simulate a larger number of patients than what is needed to reach target recruitment (ie, data amplification). On the surface, this may seem to be a mechanism to amplify the statistical power of the study and solve the problem of drawing conclusions from small studies. However, with the necessary adjustments using the combining rules described in our methodology, it has been shown that amplification does not increase statistical power for fully synthetic data as the adjusted SEs of parameter estimates are also increased [71]. The same study shows that population inferences and replicability diminish markedly without using the combining rules during the analysis of synthetic data. Further examination is needed to determine whether the same conclusions would hold for hybrid data that is only part synthetic.

The incorporation of generative models and simulated patients in industry-sponsored clinical trials would necessitate collaboration with sponsors to apply and evaluate these methods in their studies across multiple therapeutic areas to determine consistency in performance. However, recent surveys indicate that sponsors see uncertainty about regulators’ expectations and requirements for evidence as a critical barrier for the adoption of computer modeling and simulation methods in clinical trials [130]. Some efforts have identified high level principles that can be applied for the quality assurance evaluation of in silico trials [131], as well as a general good practice guidance for the application of simulations [132]. Regulators have noted the potential of simulated patients in clinical trials [133] and have further suggested adopting the Government Accountability Office accountability framework [134] for the application of machine learning models to in silico trials, which includes addressing challenges related to governance, accountability, and transparency; data considerations; and model development, performance, and validation [135]. Furthermore, synthetic data, because of their privacy-protective properties, can serve as a more readily available proxy for RWD when used in regulatory submissions [136], which can solve the data access challenge and accelerate the generation of real-world evidence. Some experts at regulatory agencies have expressed cautious optimism that synthetic data can be used by manufacturers [137], with replicability of results on real data and the need for further experimental exemplars being emphasized specifically for synthetic data generation [94]. Our study contributes to that evidence base.

Recruitment is a challenging issue not only in clinical trials but in other clinical studies as well [138,139]. These challenges are exacerbated with studies on rare diseases [140] and pediatric studies. Pediatric datasets are typically small due to a scarcity of potential study participants: there are fewer children in the population with severe disease [141]. Additional challenges include the complex ethical issues surrounding research involving children and an extra layer of consent required for pediatric participants (ie, parental consent is required in addition to patients’ assent) [142]. Many trials recruited a very small number of children [143], and studies with placebo arms are also at a disadvantage as patients are less likely to participate in case they do not get randomized to the treatment arm [144]. Therefore, the results from our study would have broader applicability beyond oncology trials in adults.

On a methodological point, this study was possible because of the ability to obtain access to the original datasets across 9 different clinical trials. While there has been strong interest in making more clinical trial data available for secondary analysis by journals, funders, the pharmaceutical industry, and regulators [145-153], data access for secondary analysis remains a challenge [154], sometimes taking many months to get data [155,156]; for example, an analysis of the success of getting individual-level data for meta-analysis projects from authors found that the percentage of the time these efforts were successful ranged from 0% to 58% [156-161]. Specifically, recent reports highlight the difficulties in accessing data for health research and machine learning analytics [162-164]. In our case, the process of getting access to all the datasets used in this study took approximately 2 years, including executing the necessary data sharing agreements and establishing collaborations with the original investigators.

One of the reasons that access to individual-level clinical trial data faces friction is concern over patient privacy by the patients and regulators [165,166]. However, the general assumption has been that synthetic data produced through generative models have low identity disclosure vulnerability because there is no unique or one-to-one mapping between the records in the synthetic data with the records in the original (ie, real) data [167-174]. While there are other types of disclosure vulnerabilities that are also relevant [175,176], some authors have argued that synthetic data can be considered nonpersonal information under statuary definitions in North America and Europe [177-179]. This would arguably be the case if disclosure vulnerability measurements demonstrate vulnerability values that are below acceptable thresholds. To that end, it is encouraging that recently study authors have been making synthetic variants of data used in their research papers publicly available to enable open science [180-183]. However, given that generating fully synthetic variants of the clinical trial datasets used in this study were not part of the original protocol, readers interested in data access can make a request to the individual data custodians, with the necessary contacts in the *Data Availability* section.

### Limitations

Although our datasets covered single site as well as multisite studies, and trials performed across multiple regions of the world with a wide range of sizes and durations, our results were obtained only from oncology trials (mostly breast cancer). There is no a priori reason for these methods not to work for other diseases and conditions, and different populations. To generalize the findings, it is necessary to replicate the findings in this work for other diseases, particularly those that impose high societal costs and where the acceleration of clinical trial evidence can be most impactful.

It is not known whether there would be evidence of a monotonic effect size over the enrollment period in other types of clinical trials and with different populations. For example, one can argue that surgery trials may exhibit a monotonic effect because surgeons become more experienced with new procedures over time. None of our studies were surgery trials. Therefore, this monotonicity relationship would need to be investigated further in these other contexts before drawing broader conclusions.

This study did not consider safety data, which tend to have fewer observations in a clinical trial. Because of the relatively smaller number of adverse events, it is more challenging to train a generative model on that kind of information. Therefore, studies comparing existing interventions or new indications would be more suited for the application of augmentation methods.

Furthermore, our retrospective analysis was limited to 9 clinical trials that were actually completed. Studies that do not reach accrual targets may have different characteristics, which could lead to different results. While our results are encouraging based on a retrospective analysis, future work should evaluate this approach through a prospective design.

When the *r* value increases, the available dataset size to train a generative model decreases. For the smallest clinical trials, in some rare instances, this resulted in generative model failure. The generative models that did not fail under those conditions had a high risk of overfitting to the training data, although they still performed better than a simple bootstrap on decision agreement. For most of our small studies (REaCT-ILIAD, REaCT–BTA, and REaCT-ZOL), the number of variables that were used for training was also quite small (8, 8, and 11, respectively; Table 1). To the extent that low dimensionality has a diluting impact on the rate of overfitting for a fixed sample size, the small number of variables would reduce that risk. Furthermore, most of the variables in some of the small trials were categorical with very few categories (ie, mostly binary), suggesting quite simple datasets were being modeled (eg, REaCT-HER2+).

We did not find that the deep learning generative models performed as well as the other approaches that were considered. One possible explanation is that additional hyperparameter tuning for these models was not performed and the default implementation model size and characteristics were used. The tuning could have improved the performance of these models. Although the default hyperparameters were suitable for low dimensional data, which was the case for the analysis datasets in many of these clinical trials.

In drawing our conclusions, we used a 0.8 value as a threshold of acceptability on CI overlap. Should one apply a more stringent threshold, then the values of *r* where the results would be acceptable would decrease further.

### Future Work

Further research is needed to provide evidence-based parameters for the number of variables that can be simulated with different types of generative models and the appropriate number of patients in a trial for training these models. This will further ensure that augmentation is not applied in inappropriate contexts.

Studies on rare diseases would have few observations, making it more challenging to train a generative model using the methods described here. Evaluations using pretrained generative models to simulate clinical trial patients should be investigated as these may be more applicable to rare disease trials.

Given that our focus was to address the insufficient accrual problem, the augmentation that was performed covered all arms of the study. However, the methods described here can be applied to individual arms as well. For example, it is possible to augment only the control arm of a trial if there were challenges accruing patients in the control arm or it could be by design to only recruit a subset of control patients and simulate the rest. Future work can evaluate such alternative augmentation strategies.

An alternative approach for augmentation to address insufficient accrual is to use pretrained generative models. For example, these models can be trained on historical clinical trial datasets, and then the pretrained models are used to simulate additional patients. This is different from the approach presented in this paper, whereby already collected data from the trial are used to train the generative models. It would be informative to compare both approaches to determine which would work better in practice, and to understand the types of historical datasets that give the best results for training these generative models.

## Appendix

Multimedia Appendix 1 Methodology details and analysis results.

## Abbreviations

ABCSG: Austrian Breast and Colorectal Cancer Study Group
BTA: bone-targeted agents
CTGAN: conditional tabular generative adversarial network
GAN: generative adversarial network
HER2+: human epidermal growth factor receptor-2 positive
REaCT: Rethinking Clinical Trials
RWD: real-world data
VAE: variational autoencoder
ZOL: zoledronate

## Data Availability

The datasets used in this study are not publicly available. Access to the datasets can be requested from the contacts listed in Multimedia Appendix 1 for each of the datasets used in this study. The synthetic data generation code is available in the *pysdg* package [184].
