# Supplementary material for: Augmenting Insufficiently Accruing Oncology Clinical Trials Using Generative Models: Validation Study
Source: J Med Internet Res. 2025 Mar 5;27:e66821. doi: 10.2196/66821 (PMC11923467; doi:10.2196/66821)
Supplement: Multimedia Appendix 1 [file jmir_v27i1e66821_app1.pdf]

Appendices

Augmenting Insufficiently Accruing Clinical Trials Using Generative Models: Validation Study

Appendix A Description of Clinical Trials

The following table summarizes each of the clinical trials that was evaluated in the study.

Trial	Description
REaCT-ILIAD (NCT02861859)	A total of 218 breast cancer patients with high risk of chemotherapy-induced nausea and vomiting (CINV) were randomized to triple therapy with added 5mg olanzapine or placebo. The primary endpoint was frequency of self-reported significant nausea [1].
REaCT-BTA (NCT02721433)	A total of 263 patients with bone metastases from breast or castration-resistant prostate cancer were randomized to 4-weekly or 12-weekly treatment using bone-targeted agents (BTAs). The dataset comprised data for 230 patients. The primary end point was change in health-related quality of life [2].
CCTG MA27 (NCT00066573)	The Canadian Cancer Trials (CCTG) group MA27 study was a large phase III trial of 7576 post-menopausal women with hormone receptor-positive early-stage breast cancer who were randomized to receive exemestane vs anastrozole. Median follow up was 4.1 years. Follow up care was semi-annually during year 1 and annually thereafter, with yearly mammogram. Event-free survival was defined as time from random assignment to time of loco-regional or distant disease recurrence, new primary breast cancer or death from any cause. In addition, this study has competing risk results making it even more applicable for global oncology practice [3].
NSABP B34 (NCT00009945)	The National Surgical Adjuvant Breast and Bowel Project (NSABP) B34 trial was a multicenter, randomized, double-blind placebo-controlled trial that enrolled 3323 with stage 1-3 breast cancer. After tumor removal, patients were stratified by age, auxiliary nodes, and oestrogen and progesterone receptor status. They were assigned to either oral clodronate daily for 3 years (n=1662) or placebo. The primary endpoint was DFS, defined as the time from random assignment to local, regional, or distant breast cancer recurrence, contralateral breast cancer, second primary malignant disease, or death from any cause before breast cancer recurrence [4]
REaCT-G/G2 (NCT02428114 & NCT02816164)	A total of 401 early breast cancer patients were randomized to receive filgrastim as primary febrile neutropenia (FN) prophylaxis. The trial evaluates whether 5 days of filgrastim was non-inferior to the 7-10 days dosing duration. The primary outcome was a composite of either FN or treatment-related hospitalization [5].
REaCT-HER2+ (NCT02632435)	A total of 48 early-stage breast cancer patients were randomized to receive trastuzumab-based chemotherapy using either peripherally inserted central catheter (PICC) or totally implanted vascular access device (PORT). The trial feasibility was evaluated through a combination of end points that mainly included patient engagement and physician engagement, both as percentages [6].

ABCSG-12 (NCT00295646)	The Australian Breast and Colorectal Cancer Study Group trial 12 (ABCSG-12) examined the effect of adding zoledronic acid in the treatment of premenopausal women with endocrine-responsive early breast cancer. A total of 1803 patients were randomized to receive goserelin plus tamoxifen or anastrozole with or without zoledronic acid for 3 years. The primary end point was disease-free survival [7].
REaCT-ZOL (NCT03664687)	A total of 211 patients with early-stage breast cancer (EBC) were randomized to receive either one dose of zoledronate or 7 doses with 6-monthly dosing for 3 years. The study was conducted to evaluate the feasibility of performing a large trial to study the effect of a single dose of zoledronate. As a primary outcome, the feasibility was assessed by a combination of metrics including activation of sites and active participation in the trial [8]
SWOG 0307 (NCT00127205)	The Southwest Oncology group (SWOG) 0307 trial was a large phase III trial of 6097 post-menopausal women with early-stage breast cancer who were randomized to receive either intravenous zoledronate, oral clodronate or oral ibandronate. Median follow up was 4.1 years at the time of the initial publication, follow up recently closed at 10 years. Follow up care was semi-annually during the first 5 years and then annually until year 10 or death, with yearly mammogram. The primary outcome was disease-free survival (DFS), defined as time from registration to first disease recurrence (local, regional, distant), new breast primary, or death from any cause. A secondary outcome was overall survival (OS), defined as time from registration to death from any cause. Patients not experiencing DFS or OS events were censored at date of last contact [9].

Table S1: A description of the nine breast cancer clinical trials.

Appendix B Published Analyses for the Nine Clinical Trials

REaCT-ILIAD study

Longitudinal data describing presence or absence of acute nausea outcome were analysed, comparing the two arms. A GEE model with logit link was used for the comparison of proportions across all visits. Estimates describes the beta coefficient, i.e. the difference of log of odds between the two arms. GEE function from GEE R package [10] was used for the analysis. Standard error of the estimate was also obtained.

REaCT-BTA study

The original analysis involving repeated measures was performed aiming to compare the overall change from baseline of the Physical Functioning component of the C30 instrument, between the two arms, across all visits. Estimates of the analysis describe the differences in mean values of the outcome (change from baseline) between the two arms, across all visits. Cancer type and study site were also used as covariates in the model.

CCTG MA27 study

Disease free survival was the primary endpoint of the study. Following the original analysis, a Cox Proportional Hazard model was used to estimate the log of the hazard ratio between the two arms. Adjustment for the main stratification variables, such as trastuzumab treatment, lymph node status and prior adjuvant chemotherapy, was also used in the model. Standard error of the estimate was also obtained.

NSABP B34 study

This study included Disease Free Survival as the primary endpoint. The primary analysis estimate was the log of the Hazard Ratio between the treatment arm (clodronate) and placebo. Cox Proportional Hazard modeling was used for the analysis, adjusting for stratifying factors, such as age, axillary nodes and oestrogen/progesterone status. Standard error was also obtained.

REaCT-G/G2 study

For the G/G2 study, original data were obtained, presenting the binary primary outcome for each cycle for each patient. A GEE model with identity link was used in order to replicate the per protocol analysis of the study and estimate the risk difference between the two arms across all chemotherapy cycles. The standard error of the estimate was also obtained. The GEE function from the GEE R package was used for the analysis [10].

REaCT-HER2+ study

For the HER2 study, aggregate data containing the number of episodes of outcomes of interest per treatment cycle for each participant were obtained. A Poisson model was used for estimating the rate of thrombosis episodes, and the rate difference between the two arms. The standard error was obtained with the use of the Delta method [11,12].

ABCSG-12 study

Disease Free Survival (DFS) was the primary endpoint of the study. The primary analysis involved the comparison of the hazard for the primary endpoint event (death from any cause or disease, whichever comes first), between the new treatment (Anastrozole) and the control arm (Tamoxifen). A co-primary analysis involved the comparison of the group who received zoledronic acid with the one who did not. Cox Proportional Hazard model was used for both analyses, with the group indicator as the single variable in the model, following the approach used in the original manuscript. Estimates of the log of hazard ratio (beta coefficient from the Cox model) and the corresponding standard error were obtained from the analysis.

REaCT-ZOL study

Descriptive statistics were obtained including estimates and standard errors of the mean values for the numerical variables, and the proportions for the categorical variables. Standard errors were estimated using asymptotic normal approximation methods.

SWOG 0307 study

The available data from the SWOG study did not include the randomization codes, and as such the original primary analysis comparing the outcomes between the two arms could not be replicated. Instead, we compared the 5-year survival probabilities between those with negative and positive/equivocal HER2 status. The estimate of the difference of survival probabilities and standard error was produced.

Appendix C Detailed Replicability Results

		Bootstrap				Sequential				Bayesian Network				CTGAN				TVAE			
Missing Ratio	Trial Short Name	Estimate Agreement	Decision Agreement	Standardized Difference	CI Overlap	Estimate Agreement	Decision Agreement	Standardized Difference	CI Overlap	Estimate Agreement	Decision Agreement	Standardized Difference	CI Overlap	Estimate Agreement	Decision Agreement	Standardized Difference	CI Overlap	Estimate Agreement	Decision Agreement	Standardized Difference	CI Overlap
0.1	ILIAD	1	1	1	0.97	1	1	1	0.99	1	1	1	0.99	1	0	1	0.96	1	0	1	0.97
	BTA	1	1	0	0.84	1	1	1	0.89	1	1	1	0.83	1	1	1	0.83	1	1	1	0.86
	CCTG	1	1	1	0.90	1	1	1	0.91	1	1	1	0.84	1	1	1	0.87	1	1	1	0.83
	NSAB	1	1	1	0.93	1	1	1	0.91	1	1	1	0.94	1	1	1	0.96	1	1	1	0.96
	G/G2	1	1	1	0.89	1	1	1	0.91	1	1	1	0.81	1	1	1	0.88	1	1	1	0.88
	HER2+	1	1	1	0.93	-	-	-	-	1	1	1	0.91	1	1	1	0.92	1	1	1	0.95
	ABCSG*	1	1	1	0.91	1	1	1	0.96	1	1	1	0.97	1	1	1	0.92	1	1	1	0.96
	ABCSG_ZOL*	1	0	1	0.91	1	0	1	0.91	1	0	1	0.90	1	0	1	0.90	1	0	1	0.92
	ZOL*	1	N/A	1	0.84	1	N/A	1	0.89	1	N/A	1	0.88	1	N/A	1	0.88	1	N/A	1	0.87
	SWOG*	1	1	1	0.93	1	1	1	0.94	1	1	1	0.92	1	1	1	0.98	1	1	1	0.89
0.2	ILIAD	1	1	1	0.88	1	1	1	0.86	1	1	1	0.92	1	1	1	0.81	1	1	1	0.91
	BTA	1	1	1	0.80	1	1	1	0.81	1	0	1	0.73	1	1	1	0.83	1	1	1	0.92
	CCTG	1	1	1	0.95	1	1	1	0.94	1	1	1	0.91	1	1	1	0.95	1	1	1	0.93
	NSAB	1	1	1	0.94	1	1	1	0.95	1	1	1	0.94	1	1	1	0.94	1	1	1	0.93
	G/G2	1	1	1	0.74	1	1	1	0.75	1	1	1	0.69	1	1	1	0.71	1	1	1	0.72
	HER2+	1	1	1	0.93	-	-	-	-	1	1	1	0.97	1	1	1	0.92	1	1	1	0.93
	ABCSG*	1	1	1	0.96	1	1	1	0.96	1	1	1	0.96	1	1	1	0.89	1	1	1	0.91
	ABCSG_ZOL*	1	0	1	0.92	1	1	1	0.94	1	0	1	0.84	1	1	1	0.93	1	1	1	0.93
	ZOL*	1	N/A	1	0.70	1	N/A	1	0.72	1	N/A	1	0.63	1	N/A	1	0.93	1	N/A	1	0.80
	SWOG*	1	1	1	0.88	1	1	1	0.90	1	1	1	0.88	1	1	1	0.91	1	1	1	0.91
0.3	ILIAD	1	0	1	0.91	1	1	1	0.89	1	1	1	0.98	1	1	1	0.82	1	1	1	0.91
	BTA	1	1	1	0.81	1	1	1	0.71	1	1	1	0.95	1	1	1	0.73	1	1	1	0.69
	CCTG	1	1	1	0.93	1	1	1	0.97	1	1	1	0.93	1	1	1	0.73	1	1	1	0.86
	NSAB	1	1	1	0.91	1	1	1	0.95	1	1	1	0.87	1	1	1	0.89	1	1	1	0.89
	G/G2	1	1	1	0.82	1	1	1	0.74	1	1	1	0.70	1	1	1	0.76	1	1	1	0.81
	HER2+	1	0	1	0.73	-	-	-	-	1	1	1	0.89	1	1	1	0.74	1	1	1	0.84
	ABCSG*	1	1	1	0.93	1	1	1	0.90	1	1	1	0.63	1	1	1	0.82	1	1	1	0.89
	ABCSG_ZOL*	1	1	1	0.92	1	1	1	0.87	1	1	1	0.94	1	1	1	0.91	1	1	1	0.86
	ZOL*	1	N/A	1	0.65	1	N/A	1	0.69	1	N/A	1	0.61	1	N/A	1	0.60	0	N/A	1	0.47
	SWOG*	1	1	1	0.82	1	1	1	0.88	1	1	1	0.78	1	1	1	0.81	1	1	1	0.68
0.4	ILIAD	1	0	1	0.88	1	1	1	0.93	1	0	1	0.93	1	0	1	0.90	1	0	1	0.89
	BTA	1	1	1	0.84	1	1	1	0.66	1	1	1	0.84	1	1	1	0.95	1	1	1	0.77
	CCTG	1	1	1	0.89	1	1	1	0.93	1	1	1	0.99	1	1	1	0.86	1	1	1	0.87
	NSAB	1	1	1	0.73	1	1	1	0.69	1	1	1	0.81	1	1	1	0.71	0	0	1	0.40
	G/G2	1	1	1	0.73	1	1	1	0.63	1	1	1	0.67	1	1	1	0.82	1	1	1	0.69
	HER2+	1	0	1	0.65	-	-	-	-	1	1	1	0.93	1	1	1	0.83	1	1	1	0.85
	ABCSG*	1	1	1	0.89	1	1	1	0.87	1	1	1	0.91	1	1	1	0.71	1	1	1	0.85
	ABCSG_ZOL*	1	1	1	0.89	1	1	1	0.90	1	1	1	0.91	1	0	1	0.75	1	1	1	0.84
	ZOL*	1	N/A	1	0.67	1	N/A	1	0.67	1	N/A	1	0.62	1	N/A	1	0.87	0	N/A	1	0.33
	SWOG*	1	1	1	0.89	1	1	1	0.91	1	1	1	0.89	1	1	1	0.86	1	1	1	0.79
0.5	ILIAD	1	0	1	0.85	1	0	1	0.92	1	0	1	0.84	1	0	1	0.87	1	0	1	0.76
	BTA	1	1	1	0.84	1	1	1	0.68	1	1	1	0.76	1	1	1	0.95	1	1	1	0.93
	CCTG	1	1	1	0.87	1	1	1	0.71	1	1	1	0.79	1	1	1	0.86	1	1	1	0.75
	NSAB	1	1	1	0.76	1	1	1	0.88	1	1	1	0.84	1	1	1	0.74	1	1	1	0.73
	G/G2	1	1	1	0.64	0	1	1	0.40	1	1	1	0.61	1	1	1	0.79	1	1	1	0.75
	HER2+	1	0	1	0.63	-	-	-	-	-	-	-	-	-	-	-	-	-	-	-	-
	ABCSG*	1	1	1	0.86	1	1	1	0.89	1	1	1	0.80	1	1	1	0.87	1	1	1	0.82
	ABCSG_ZOL*	1	1	1	0.86	1	1	1	0.85	1	1	1	0.77	1	1	1	0.81	1	1	1	0.81
	ZOL*	1	N/A	1	0.80	1	N/A	1	0.93	1	N/A	1	0.51	1	N/A	1	0.86	1	N/A	1	0.93
	SWOG*	1	1	1	0.86	1	1	1	0.87	1	1	1	0.85	1	1	1	0.89	1	1	1	0.89

¥ The original study is descriptive and does not include any arms. Hence, the decision agreement does not apply.

* Do not have stratification information, so basic non-stratified augmentation is used.
§ The original study does not include any arms. However, the conducted analysis compares the survival probability at 5 years for those with and without HER+. The decision agreement is calculated accordingly.
- The generative model failed due to small sample size. In the case of sequential decision it is by design in the implementation not to train a model with less than 50 observations.

Table S2: Agreement, standardized difference, and CI overlap results for results up to r=0.5.

Appendix D Data Availability

Access to the datasets can be requested from the following contacts for each of the datasets used in this study.

Dataset	Contact
REaCT-ILIAD	Dr. Mark Clemons,<br>Institution: Ottawa Hospital Research Institute
REaCT-BTA	Dr. Mark Clemons<br>Institution: Ottawa Hospital Research Institute
CCTG MA27	Dr. Paul Goss<br>Institution: Canadian Cancer Trials Group
NSABP B34	Norman Wolmark<br>Institution: NSABP Foundation Inc
REaCT-G/G2	Dr. Mark Clemons<br>Institution: Ottawa Hospital Research Institute
REaCT-HER2+	Dr. Mark Clemons<br>Institution: Ottawa Hospital Research Institute
ABCSG-12	Raimund Jakesz<br>Institution: Austrian Breast & Colorectal Cancer Study Group
REaCT-ZOL	Dr. Mark Clemons<br>Institution: Ottawa Hospital Research Institute
SWOG 0307	Julie R. Gralow<br>Institution: SWOG network within National Cancer Institute

References

1 Clemons M, Dranitsaris G, Sienkiewicz M, *et al.* A randomized trial of individualized versus standard of care antiemetic therapy for breast cancer patients at high risk for chemotherapy-induced nausea and vomiting. *Breast*. 2020;54:278–85. doi: 10.1016/j.breast.2020.11.002

2 Clemons M, Ong M, Stober C, *et al.* A randomised trial of 4- versus 12-weekly administration of bone-targeted agents in patients with bone metastases from breast or castration-resistant prostate cancer. *Eur J Cancer*. 2021;142:132–40. doi: 10.1016/j.ejca.2020.08.019

3 Goss PE. Exemestane versus anastrozole in postmenopausal women with early breast cancer: NCIC CTG MA.27–a randomized controlled phase III trial. *J Clin Oncol*. 2013;31:1398-1404,. doi: 10.1200/JCO.2012.44.7805.

4 Paterson AHG, Anderson SJ, Lembersky BC, *et al.* Oral clodronate for adjuvant treatment of operable breast cancer (National Surgical Adjuvant Breast and Bowel Project protocol B-34): a multicentre, placebo-controlled, randomised trial. *Lancet Oncol*. 2012;13:734–42. doi: 10.1016/S1470-2045(12)70226-7

5 Clemons M, Fergusson D, Simos D, *et al.* A multicentre, randomised trial comparing schedules of G-CSF (filgrastim) administration for primary prophylaxis of chemotherapy-induced febrile neutropenia in early stage breast cancer. *Ann Oncol*. 2020;31:951–7. doi: 10.1016/j.annonc.2020.04.005

6 Clemons M, Stober C, Kehoe A, *et al.* A randomized trial comparing vascular access strategies for patients receiving chemotherapy with trastuzumab for early-stage breast cancer. *Support Care Cancer*. 2020;28:4891–9. doi: 10.1007/s00520-020-05326-y

7 Gnant M, Mlineritsch B, Schippinger W, *et al.* Endocrine therapy plus zoledronic acid in premenopausal breast cancer. *N Engl J Med*. 2009;360:679–91. doi: 10.1056/NEJMoa0806285

8 Awan A, Ng T, Conter H, *et al.* Feasibility outcomes of a randomised, multicentre, pilot trial comparing standard 6-monthly dosing of adjuvant zoledronate with a single one-time dose in patients with early stage breast cancer. *J Bone Oncol*. 2021;26:100343. doi: 10.1016/j.jbo.2020.100343

9 Gralow JR. Phase III Randomized Trial of Bisphosphonates as Adjuvant Therapy in Breast Cancer: S0307. *J Natl Cancer Inst*. 2020;112:698-707,. doi: 10.1093/jnci/djz215.

10 Carey VJ, and 4.4) TSL (R port of versions 3 13, src/d*) CM (LINPACK routines in, *et al.* gee: Generalized Estimation Equation Solver. 2022.

11 Oehlert GW. A Note on the Delta Method. *The American Statistician*. 1992;46:27–9. doi: 10.1080/00031305.1992.10475842

12 Ver Hoef JM. Who Invented the Delta Method? *The American Statistician*. 2012;66:124–7. doi: 10.1080/00031305.2012.687494
